# Anterolateral Ligament Reconstruction Combined with Anterior Cruciate Ligament Reconstruction: Clinical and Functional Outcomes

**DOI:** 10.3390/medicina61061011

**Published:** 2025-05-28

**Authors:** Giuseppe Danilo Cassano, Lorenzo Moretti, Michele Coviello, Ilaria Bortone, Mariapia Musci, Ennio Favilla, Giuseppe Solarino

**Affiliations:** 1 Orthopaedic and Trauma Unit, Department of Basic Medical Sciences, Neuroscience and Sense Organs, School of Medicine, AOU Consorziale Policlinico, University of Bari “Aldo Moro”, Piazza Giulio Cesare 11, 70124 Bari, Italy; lorenzomoretti1@gmail.com (L.M.); ilariabortone@gmail.com (I.B.); enniofavilla@gmail.com (E.F.); giuseppe.solarino@uniba.it (G.S.); 2 Orthopaedics Unit, Department of Clinical and Experimental Medicine, Faculty of Medicine and Surgery, University of Foggia, Policlinico Riuniti di Foggia, 71122 Foggia, Italy; michelecoviello91@gmail.com; 3 Department of Mechanical Mathematics and Management (DMMM), Politecnico di Bari, 70126 Bari, Italy; pia19091997@gmail.com

**Keywords:** anterior cruciate ligament reconstruction, ACL, anterolateral ligament reconstruction, combined procedure, ALL, clinical evaluation, functional outcomes, biomechanical tests

## Abstract

*Background and Objectives*: The anterior cruciate ligament (ACL) is crucial for knee stability, preventing anterior displacement of the tibia and rotation relative to the femur. Despite ACL reconstruction (ACLR), residual instability is common, affecting knee function. Anterolateral ligament reconstruction (ALLR) alongside ACLR improves outcomes, as the ALL plays a significant role in rotational stability. This study aims to assess the clinical and functional outcomes of the ACLR+ALLR combination using biomechanical testing in patients with at least ten months of follow-up. *Materials and Methods:* This cross-sectional comparative cohort study involves patients with ACLR. Inclusion criteria were adult patients who underwent ACLR within the last 3 years, with the same surgical technique performed by a single operator. Patients underwent anamnestic and clinical evaluation and completed Lysholm and KOOS questionnaires. Biomechanical tests included a Unilateral Drop Jump, a Countermovement Jump with knee rotation, and a five-repetition Sit-To-Stand. Force platforms, a camera, and surface electromyography were used to assess biomechanical stability and joint function. *Results:* This study included 18 subjects, 5 with ACLR and ALLR, and 13 with ACLR alone. The groups showed no significant differences in the KOOS and Lysholm scales and clinical outcomes. Muscle trophism reduction compared to the contralateral limb was noted in both groups. Biomechanical evaluations showed no difference in Quadriceps muscle activity during the landing phase of the Drop Jump. However, the ACL-ALL group exhibited fewer spikes and fewer knee joint angular excursions during ground impact stabilization. In the 5-STS task, a significant difference was observed in the vertical force peak. Differences in muscle activity during foot rotation and force components during the jumping phase were noted in the Countermovement Jump. *Conclusions:* ACLR combined with ALLR shows similar perceived joint function but improved biomechanical joint stability. Further studies with larger samples and longer follow-ups are needed for validation.

## 1. Introduction

The anterior cruciate ligament (ACL) and the posterior cruciate ligament (PCL) constitute the knee’s central pivot. The ACL plays a fundamental role in the stability of this joint by limiting the anterior displacement of the tibia and its rotation relative to the femur.

Following a knee sprain that results in an ACL injury, the ACL’s reconstruction is paramount to ensure optimal joint stability and adequate return to pre-injury levels of daily and athletic activity [1].

However, many patients who undergo ACL reconstruction (ACLR) present with residual instability, as demonstrated by dynamic stability testing (pivot-shift test) and incomplete recovery of knee function after surgery [1].

Knee sprains can result in peripheral capsular ligament injuries. Anterolateral compartment injury in combination with ACL injury results in a grade 3 pivot shift [2], highlighting the crucial role of the anterolateral capsular ligamentous structures of the knee in providing increased rotational stability. ACL reconstruction related to anterolateral ligament reconstruction (ALLR) provides better outcomes than ACL reconstruction alone [3].

The anterolateral ligament (ALL) is a structure whose origin is located at the prominence of the lateral femoral epicondyle, with an oblique course, solid attachments to the lateral meniscus, and an insertion on the anterolateral tibia located midway between Gerdy’s tubercle and the head of the fibula. The ALL tenses when the tibia is translated forward and intrarotated [4,5,6]. A biomechanical study on human cadaveric knees showed that the ALL is an important stabilizer of anteroposterior translation and internal rotation of the tibia; reconstruction of an injured ALL, combined with ACLR, significantly improved joint stability [1]. Through progressive dissection of the ACL and ALL and biomechanical analysis of the knee, it has been shown that axial rotation is mainly increased in combined injuries compared to ACL injuries alone [7].

ACLR combined with ALLR was widely studied, and the advantages of this type of surgery emerged. Sonnery-Cottet et al. [3] have provided an overview of the most relevant research on the ALL and presented the consensus of the ALL Expert Group on the anatomy, radiographic landmarks, biomechanics, clinical and radiographic diagnosis, lesion classification, surgical technique, and clinical outcomes. From their consideration, it emerged that persistent rotational instability, indicated by a positive pivot shift, may be present in up to 25% of cases after an isolated intra-articular ACL reconstruction procedure, and persistent rotational instability is a risk factor for new re-injuries. Hyperlaxed patients have an increased risk of presenting a persistent pivot shift. So, improvement of rotational stability is mandatory for these patients. For these reasons, the goals of a combined ACL and ALL reconstruction are to reduce the rate of ACL re-injury recurrence and to improve rotational stability control of the knee. Therefore, the ALL-study group has identified major and minor criteria to associate ALL with ACL reconstruction [3]. In a study with more than eight years of follow-up, the same authors compared 86 matched pairs of patients who underwent ACLR or ACLR plus ALLR. The results showed that patients who underwent ACLR plus ALLR had significantly better long-term ACL graft survival, lower overall re-intervention rates, and no increase in complications compared to patients who underwent isolated ACLR. In addition, patients who underwent isolated ACLR had a five times increased risk of revision surgery [8].

Different techniques, anatomical and non-anatomical, for reconstructing the ALL have been described [9,10,11,12], and even at long follow-ups, do not appear to increase the risk of osteoarthritis when compared to ACLR alone [13].

However, there are no studies in the literature that have evaluated these patients from a biomechanical standpoint.

This study aims to evaluate clinical and functional outcomes of ACL reconstruction combined with ALL reconstruction, through biomechanical testing, in patients with at least ten months of follow-up.

## 2. Materials and Methods

This is a cross-sectional comparative cohort study. The patients were recruited from the UOC Orthopaedics and Traumatology of the “Azienda Ospedaliero Universitaria Consorziale del Policlinico di Bari”. Ethical clearance was obtained from our local ethical committee (prot n.0076727-16 October 2020), and all patients gave informed consent before enrollment.

The inclusion criteria were (1) adult patients (between 18 and 45 years of age); (2) ACL reconstruction surgery within the last three years; (3) a single operator performing the surgery; and (4) the same surgical technique used for ACL reconstruction. The exclusion criteria were (1) neo-lesion of the ACL following new distortive trauma; (2) refusal to participate in this study; and (3) RAMP or ROOT sutures or subtotal meniscectomies due to possible alteration of joint biomechanics.

The patients were called back to the Functional Assessment Laboratory of Bari General Hospital and underwent an anamnestic examination, clinical evaluation, and administration of Lysholm [14] and KOOS [15] questionnaires. The follow-up time was variable among the patients; in fact, even though the inclusion criterion was that they had been operated on within the previous three years, the patients included in this study had a follow-up time ranging between two years and one and a half years. The sample was divided into two groups: group A patients undergoing ACLR, and group B patients undergoing ACLR and ALLR.

### 2.1. Surgical Technique

Patients were operated on under spinal anesthetic treatment by the same experienced knee surgeon (L.M.), and in both groups, the ACLR all-inside technique with a quadruple semitendinosus graft (ST4), as described by Cerulli et al. [16], was performed.

A 110° femoral aimer (Femoral curved ACL Marking Hook, Arthrex©) and a 55° tibial aimer (Tibial ACL Marking Hook, Arthrex©) were pointed to the anatomical ACL footprints at the 10 o’clock position under direct arthroscopic view. The retrograde femoral and tibial half tunnels using FlipCutter^®^ II Drill (Arthrex©) were created; they measured about 2.5 cm. The graft was fixed with the knee in extension with a cortical suspension system (Tight-Rope, Arthrex©) on both the femoral and tibial sides.

In group B, after ACLR, anatomic ALLR was performed as described by Chahla [9]. The Gracilis muscle tendon was harvested and prepared by suturing it at the ends. Isometric tension points are identified at the femoral and tibial levels and two half tunnels were created with a 7 mm burr at the tibial level and 4.5 mm at the femoral level for a length of approximately 2 cm. Using hemostatic forceps, the graft was passed through a passage pocket under the iliotibial band and locked at the femoral and tibial level with two Swivelock anchors (Arthrex©, Naples, FL, USA).

### 2.2. Biomechanical Test

**Figure 1:**
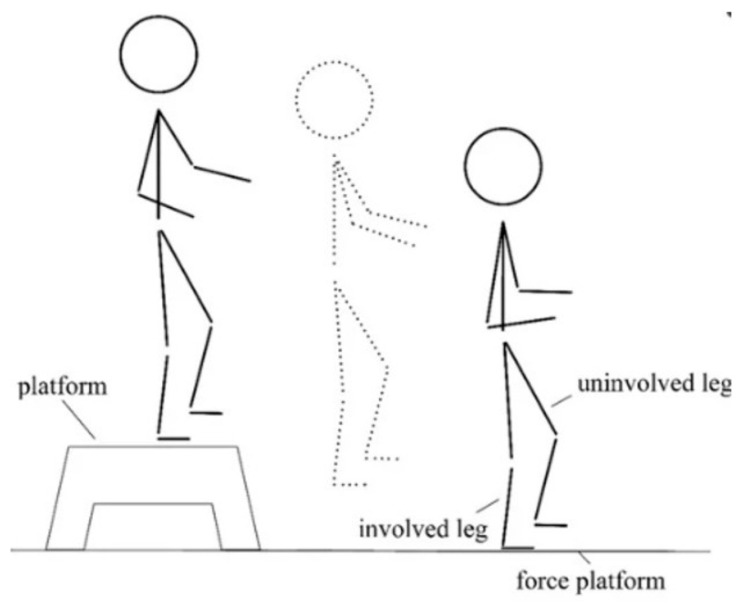
Schematic illustration of the Unilateral Drop Jump technique.

**Figure 2:**
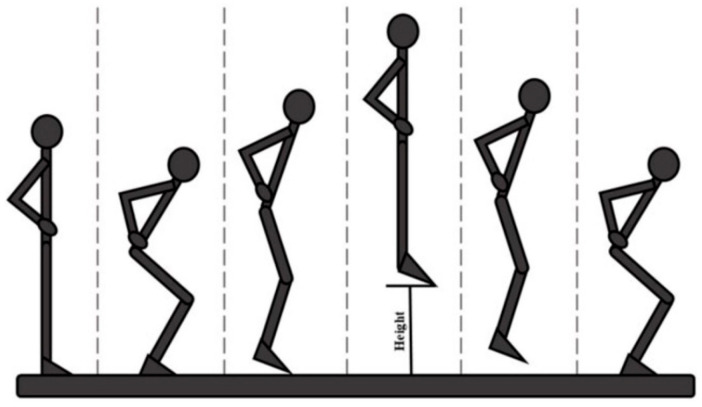
Schematic illustration of the Countermovement Jump technique, step by step.

Biomechanical assessments were performed with the aid of the BTS Smart Performance System (BTS S.p.A., Milano, Italy), consisting of force platforms, cameras, and wireless surface electromyography (sEMG) probes placed on the Vastus Medialis (VM) and Vastus Lateralis (VL) of both legs. The participants performed the following three tasks in the following order:

- Unilateral Drop Jump (Figure 1) [17] in which the subject stands on a 30 cm high platform. At the therapist’s start, the subject had to drop, with one limb, onto the force platform in front of the step, stabilize, and remain there until the therapist’s stop warning. This test assesses the strength of the lower limbs (performed first with the healthy limb and then with the limb undergoing surgery).
- Countermovement Jump (Figure 2) [18] with the change in knee direction: this consists of a jump squat performed from a standing position, with both feet positioned on the force platforms. At the end of the traditional exercise, our protocol included the execution of an internal rotation of the knee so that the foot is rotated approximately 90° from its natural position (performed first with the healthy limb and then with the operated limb).
- The five-repetition Sit-To-Stand (5-STS) test is a widely used clinical assessment for detecting motor problems [19]. The subject had to stand up and sit down from the chair five times, starting from a seated position with arms crossed over the chest so that only the lower limbs were used to perform the movement.

Methodological controls were implemented to minimize EMG variability. The order of tasks was randomized for each participant, and adequate rest periods were allowed between tests to avoid fatigue-related changes in EMG activity.

The selected tasks proved to be dynamic and comprehensive tests useful in evaluating recovery after ACL reconstruction [20,21,22,23]. They allow the assessment of several key parameters concerning strength, stability, and neuromuscular control of the operated limb, which are fundamental elements for predicting the return to sport and preventing the risk of recurrence. These tasks make it possible to compare the reconstructed limb’s absorption and force production capacity and to observe any compensation or abnormal motor patterns that the patients might develop to protect the operated limb. These patterns are fundamental to be analyzed because they may increase the risk of new injuries to the knee or other joints.

### 2.3. Biomechanical Data Processing

For each motor task performed by the study participants, different variables were extracted. The force platforms, described above, were used to extract data on movement kinetics; surface electromyography probes were used to obtain information on muscle activation during task execution. Finally, the camera was useful for conducting video analyses.

The same experienced assessor conducted all testing sessions, including participant instruction, electrode placement, and task execution supervision. This approach was useful to ensure consistency in data collection procedures and to minimize inter-assessor variability.

The first step to obtain these variables is the pre-processing phase, which first allows the extraction of signals from the SMART Sportlab Clinic software (BTS S.p.A., Milano, Italy) and the subsequent processing of biomechanical data in Matlab 2022a.

The pre-processing phase also included signal segmentation to identify the beginning and end of the motor task using the videos obtained with the VIXTA camera (BTS S.p.A., Milano, Italy).

The electromyographic signal was initially filtered using a 4th-order Butterworth bandpass filter with cut-off frequencies of 10 Hz and 450 Hz. The signal was further processed to obtain the envelope, using a Butterworth 2nd-order low-pass filter with a cut-off frequency of 6 Hz. Muscle activation was calculated using the method of Hodges and Bui [24]. Muscle activity was measured as the muscle’s duration of activation, considered during the task or task phase. The Right Vastus Lateralis (RVL), Left Vastus Lateralis (LVL), Right Vastus Medialis (RVM), and Left Vastus Medialis (LVM) muscles were the muscles whose electromyographic signal was acquired during the three tasks.

For the kinetic analysis, the vertically exchanged force between the foot and the ground, i.e., the y-component of the force signal, was analyzed. The latter was filtered with a 4th-order low-pass Butterworth filter with a cutoff frequency of 15 Hz.

The Drop Jump task video analysis was performed with Tracker 6.1.3 software (https://physlets.org/tracker/ accessed on 1 May 2023) to measure the range of motion of the knee joint during the movement. After uploading the video, the software was calibrated using the dimensions of the force platforms, the origin of the reference system, and the reference points (at the epicondyles and malleolus) were defined. The coordinates of the reference points (x and y components) were saved and extracted into text files for further processing in Matlab 2022a. The coordinates of the points defining the knee and ankle position in time were used to obtain the knee angle.

For the biomechanical analysis of the Drop Jump exercise, performed with the operated limb (o) and the unoperated limb (h), a total of 26 features were extracted:

- From the sEMG data the duration of muscle activation (preATT_OVL_o, preATT_HVL_o, preATT_OVM_o, preATT_HVM_o, preATT_OVL_h, preATT_HVL_h, prerATT_OVM_h, preATT_HVM_h) and the maximum envelope (env_OVL_o, env_HVL_o, env_OVM_o, env_HVM_o, env_OVL_h, env_HVL_h, env_OVM_h, env_HVM_h) during the entire exercise for the 4 muscles, for a total of 16 muscle variables. To better understand the significance of the variables, it is specified that “O” or “H” refers to the muscle of the operated limb (O) or the contralateral (healthy) limb (H);
- From the kinetic data obtained from the force platforms, the vertical force peak (VGRF_o, VGRF_h), the number of vertical force peaks recorded at landing (NPTS_Fy_o, NPTS_FY_h), the off-axis parameter [25] indicating how much of the vertical component of the force was distributed over the horizontal and transverse component to stabilize the landing (Off-axis_x_o, Off-axis_z_o, Off-axis_x_h, Off-axis_z_h) were obtained for a total of 8 variables;
- From the video analysis, the range of motion [26] (ROM_o, ROM_h) of the knee joint was extracted using videos obtained during the execution of the task performed.

The analysis of the Countermovement Jump exercise with knee direction rotation resulted in a total of 40 variables. The exercise was repeated: first, the jump was performed with subsequent foot rotation of the non-operated limb (h) and then of the operated limb (o). Consequently, the task was divided into a jumping phase (1) and a knee rotation phase (2);

- From the processing of the electromyographic signal, the envelope peak (peak_envOVL_1o, peak_envOVL_2o, peak_envHVL_1o, peak_envHVL_2o, peak_envOVM_1o, peak_envOVM_2o, peak_envHVM_1o, peak_envHVM_2o, peak_envOVL_1h, peak_envOVL_2h, peak_envHVL_1h, peak_envHVL_2h, peak_envOVM_1h, peak_envOVM_2h, peak_envHVM_1h, peak_envHVM_2h) and the duration of muscular activation (onset_OVL_1o, onset_OVL_2o, onset_HVL_1o, onset_HVL_2o, onset_OVM_1o, onset_OVM_2o, onset_HVM_1o, onset_HVM_2o, onset_OVL_1h, onset_OVL_2h, onset_HVL_1h, onset_HVL_2h, onset_OVM_1h, onset_OVM_2h, onset_HVM_1h, onset_HVM_2h) were calculated during each phase of the task for the 4 muscles, listed above, resulting in 32 variables. The letters “O” or “H” refer to the muscle of the operated limb (O) or the contralateral (healthy) limb (H);
- From a kinetic point of view, the vertical force peak (GRF_1o, GRF_2o, GRF_1o, GRF_2o) was derived, resulting in 4 variables.

For the 5-repetition Sit-To-Stand test, 13 variables were obtained. Each variable was calculated for each repetition of the stand-up and sit-down cycle.

- From the analysis of the electromyographic signal, the peak of the envelope (max_env_OVL, max_env_HVL, max_env_OVM, max_env_HVM) and the duration of muscle activation during exercise (att_OVL, att_HVL, att_OVM, att_HVM) were extracted for a total of 8 features. The letters “O” or “H” refer to the muscle of the operated limb (O) or the contralateral (healthy) limb (H).
- The analysis of the force exchanged with the ground made it possible to obtain the duration of the entire exercise (duration_STS), the average duration of the lift–seat cycles (dur_cycles_mean), the peak vertical force (peak_F_rise) for the ascent phase alone, the time required to reach this peak (t_peak), and the average vertical force during each cycle (mean_F_rise) for the ascent phase alone for a total of 5 variables.

### 2.4. Statistical Analysis

Data were collected and analyzed using Microsoft Excel and RStudio^®^. R version 4.2.3. Categorical variables were presented as numbers or percentages. Continuous variables were presented as mean and standard deviation. The Shapiro–Wilk test was conducted to verify the normal distribution of the data. The Mann–Whitney U test (Wilcoxon rank sum) was used to compare the mean scores between groups. The effect size (Cohen’s d) was used as a measure of the magnitude of the difference between the groups. Data presented in this study are available upon request from the corresponding author.

## 3. Results

The present study population consisted of 18 subjects with an average age of 24 years, of whom 5 had ACLR and ALLR (age 22.4 ± 1.94) and 13 with ACLR alone (age 25.2 ± 2.29). Clinically, there were no major differences between the two groups. Both groups of patients who underwent ACL reconstruction only and those who underwent ACL-ALL achieved high scores on the KOOS (85 in the ACL group and 87 in the ACL-ALL group) and Lysholm scales (90 in the ACL group and 88 in the ACL-ALL group). In terms of muscle trophism, there were also no anthropometric differences between the two groups. In both groups, there was a reduction in muscle trophism compared to the unoperated contralateral limb. The characteristics of the populations are shown in Table 1. There were no statistically significant differences between the two groups in terms of sociodemographic and clinical variables.

We then compared the variables extracted from the biomechanical evaluations in the two groups. In the landing phase of the Drop Jump, the activity of the quadriceps muscle did not differ between the two groups. On the other hand, a difference was observed in the stabilization capacity following ground impact (NPTS_Fy): the ACL-ALL group showed fewer spikes and less angular excursion of the knee joint, as shown in Table 2.

In the 5-repetition Sit-To-Stand task, as shown in Table 3, a significant difference (at the low 0.37 limit) was only observed in the peak of the vertical force for the ascent phase.

The Countermovement Jump is divided into two phases: the jump phase (1) and the knee rotation phase (2).

The differences, as shown in Table 4, were observed in terms of muscle activity for the side operated in the knee rotation phase; in terms of the force component, the differences were found in the jumping phase.

## 4. Discussion

The ACL reconstruction in patients with hyperlaxity, athletes frequently involved in pivot movements and revision surgeries, and patients with a grade > 2 pivot shift should be associated with ALL reconstruction to ensure optimal rotational and translational stability, since ACL reconstruction alone would not allow this. Therefore, associating ALLR would guarantee better outcomes and a lower rate of re-injury [8].

The objective of this work was to evaluate the differences in KOOS and LYSHOLM clinical scores and biomechanical assessments in patients undergoing ACL reconstruction and ALL reconstruction compared to patients undergoing ACL reconstruction alone.

To achieve this objective, 18 patients belonging to the Orthopedic Clinic of the University Hospital of Bari were identified according to specific inclusion and exclusion criteria and enrolled in this study. After providing consent, the patients underwent anamnestic, clinical, and biomechanical evaluations to assess any differences in response. The selected biomechanical tests were identified by their relevance in assessing postural stability, physical performance, reactivity and elasticity of the lower limbs, and the ability to massively stress the knee joint to verify its stability [27].

Clinically, no significant differences were observed in the KOOS and LYSHOLM scores: all patients showed near-optimal recovery of perceived joint function in daily life.

From the biomechanical point of view, some differences emerged in muscle activation and force profiles between the two groups. In the Drop Jump test, i.e., when landing on the force plate from a height of approximately 30 cm, a reduction in the number of spikes was noted in the ACL-ALL group compared to the ACL group, and a reduction in joint ROM excursion in the landing phase. These results could mean that the knee with the ALL reconstruction is more stable. In previous studies, Brophy et al. [28] demonstrated, only considering the ACL reconstruction, that dynamic stability significantly improved up to 12 months after the operation. They noted that improvement in stability occurred in the medial/lateral and anterior/posterior planes of motion. Moreover, recovery of knee stability following ligament reconstruction depends on adequate surgical reconstruction and a rehabilitation program to recover proprioceptive control of the knee [29]. It is important to underline that, in our study, the rehabilitation protocol was not performed at a single location under our supervision, so the patients may not have been followed as precisely as prescribed. For this reason, we cannot determine if the stability, after the follow-up time, returned to preinjury levels. This difference in post-operative rehabilitation may cause some bias.

On the other hand, Schon et al. [30] demonstrated that the anatomic ALLR combined with ACLR significantly reduced rotatory laxity of the knee beyond 30° of knee flexion. However, they noted that a significant over-constraint occurred after ALLR, regardless of fixation angle.

If we consider the kinetics of movement assessed with the 5-repetition Sit-To-Stand test, no differences emerged between groups except for the peak of the vertical force during the ascent phase. This could mean that, as we would have expected, in the kinetics performed without rotations or joint stability stress, there are no differences if the ALL reconstruction is associated. The significant difference at the low limits (0.37) in the peak of the vertical force could be explained as a compensation mechanism, as the Gracile muscle tendon was removed in that limb. Different studies [31,32] demonstrated that hamstring tendons regenerated after the harvest of gracilis tendons for ACL reconstruction. Considering this, our result shows a clinically relevant finding: patients may be informed about the possibility of the occurrence of compensatory mechanisms that have to be carefully examined in the rehabilitation protocol.

Furthermore, considering the last Countermovement Jump and change in knee direction, the group of patients who underwent ACL and ALL reconstruction showed prolonged muscle activation but lower intensity. Other studies have assessed muscle activity using sEMG, i.e., Coats-Thomas et al. [33] found differences as a function of ACL reconstruction status and sex for the Quadriceps, Hamstring, and Gastrocnemius muscles during a jump-unanticipated cut maneuver. It is important to underline the later peak timing in the Rectus Femoris, Vastus Medialis, Biceps Femoris, and Medial Gastrocnemius of the reconstructed ACL compared to the healthy ACL, and the Hamstring and Quadriceps muscles peaked earlier in females than in males. While we obtained results relative to muscle activation, Zunzarren et al. [34] demonstrated that at a follow-up time of 3 years after ACL reconstruction, the neuromuscular activation deficit was high (roughly 42%). The deficits are related to the entire limb and not only to the Hamstring. These considerations could lead to a better understanding of the importance of an appropriate rehabilitation protocol.

Considering the change in direction of the knee phase of the Countermovement Jump test, the variables analyzed, and the activation peaks obtained leads us to hypothesize that the patients who underwent ACLR and ALLR are characterized by greater articular stability of the knee. We have demonstrated that the ACLR and ALLR combination allows major stability of the knee both during the Drop Jump and the Countermovement Jump. Delaloye et al. [35] found that, contrary to isolated ACLR, in knees where combined ACLR and ALLR were performed, the original knee stability was restored in anterior translation and internal rotation. In addition, ALLR was demonstrated to be a better technique in terms of restoring knee kinematics, and it did not overconstrain the knee.

The results of the research could lead to practical implications in clinical and rehabilitation practice. The results obtained could be used to enhance the existing treatment protocols, to develop patient-specific procedures for particular subjects such as athletes in pivot-heavy sports (e.g., soccer or basketball). Furthermore, rehabilitation procedures can be developed with a greater focus on rotational control and proprioception to optimize recovery and allow patients to return to daily or sport-related activities.

Despite our research findings, the study presents some limitations: a small sample size and a short follow-up period that can affect how the results are generalizable and long-lasting. This study’s lack of diversity in patient demographics (i.e., gender) and exercise level could also impact how broadly the findings can be applied. The possible variation in the post-operative rehabilitation protocols is another variable that can affect the results.

## 5. Conclusions

Patients who underwent ACL reconstruction and ALL compared to patients who underwent ACL reconstruction alone have a similar perceived joint function in daily life, while biomechanical evaluations show greater joint stability. A key strength of this study is the use of biomechanical evaluation, which, combined with the patient’s subjective physical function assessment, provides quantitative and objective data on joint stability and strength. This allows a deeper understanding of the surgical result using a more accurate assessment of the reconstructed joint’s performance under load and during dynamic movement. The objective data are essential for detecting subtle variations in joint behavior that might not be noticeable to patients but may impact long-term joint health and re-injury risk. This study presents some limitations: a small sample size and a short follow-up period. These characteristics can affect how the results are generalizable and long-lasting. This study’s lack of diversity in patients could also impact how broadly the findings can be applied. The possible variation in the post-operative rehabilitation protocols is another variable that can affect the results and add confounding variables. Further studies with larger sample numbers and longer-term follow-ups are needed to confirm the obtained data and to standardize biomechanical assessment scores for joint strength and stability.

## Figures and Tables

**Table 1:** Socio-demographic and clinical characterization of patients in groups.

Variable	ACL		ACL-ALL		
	Median	IQR	Median	IQR	Effect Size
Age [years]	24	11	18	11	0.20 (−0.15–0.45)
Distance to surgery [months]	13	21	10	2	0.18 (−0.21–0.45)
KOOS	85	18	87	7	0.07 (−0.31–0.21)
LYSHOLM	90	23	88	5	0.14 (−0.20–0.37)
TROPHISM (Operated Side) [cm]	45	4	44	1	0.20 (−0.17–0.47)
TROPHISM (Healthy Side) [cm]	46	6	46	1	0.16 (−0.20–0.39)

KOOS: Knee Injury and Osteoarthritis Outcome Score; LYSHOLM: Lysholm Knee Scoring Scale; TROPHISM: thigh muscle trophism.

**Table 2:** Characterization of biomechanical variables during the Drop Jump test.

Variable	ACL		ACL-ALL			
	Median	IQR	Median	IQR	Effect Size	
preATT_OVL_o	0.09	0.19	0.23	0.27	**0.48 (0.17–0.8)**	**moderate**
preATT_HVL_o	1.07	0.54	1.11	0.28	0.11 (−0.24–0.31)	
preATT_OVM_o	0.09	0.14	0.29	0.09	**0.38 (0.04–0.72)**	**moderate**
preATT_HVM_o	1.27	0.74	1.09	0.86	0.01 (−0.41–0.11)	
preATT_OVL_h	1.05	0.86	1.11	0.54	0.01 (−0.38–0.1)	
preATT_HVL_h	0.12	0.31	0.25	0.14	0.04 (−0.36–0.15)	
preATT_OVM_h	0.81	0.41	0.81	0.69	0.04 (−0.36–0.16)	
preATT_HVM_h	0.22	0.22	0.35	0.36	0.04 (−0.42–0.17)	
env_OVL_o	187.39	191.89	141.95	87.85	0.01 (−0.4–0.11)	
env_HVL_o	47.68	55.08	49.63	64.58	0.14 (−0.19–0.36)	
env_OVM_o	227.68	92.7	187.24	162.09	0.19 (−0.18–0.46)	
env_HVM_o	87.11	156.02	38.67	75.59	0.09(−0.27–0.27)	
env_OVL_h	56.96	49.15	69.45	58.71	0.12 (−0.23–0.31)	
env_HVL_h	215.8	170.72	152.83	32.72	0.27 (−0.09–0.61)	
env_OVM_h	59.24	59.83	62.82	33.89	0.01 (−0.38–0.1)	
env_HVM_h	318.22	225.69	247.01	114.88	0.30 (−0.06–0.61)	
VGRF_o	1617.018	583.39	1437.221	328.35	0.27 (−0.07–0.59)	
VGRF_h	1833.156	514.46	1742.171	380.10	0.19 (−0.15–0.46)	
NPTS_Fy_o	4	3	1	1	0.45(0.11–0.8)	**moderate**
NPTS_Fy_h	5	4	2	0	0.49(0.16–0.84)	**moderate**
ROM_o	44.36	11.58	35.69	5.65	0.51(0.21–0.82)	**large**
ROM_h	47.86	14.80	43.49	6.92	0.35 (−0.0043–0.69)	
Off-axis_x_o	2.87	3.73	1.99	5.71	0.14 (−0.24–0.38)	
Off-axis_z_o	2.96	1.80	3.59	2.16	0.30 (−0.03–0.61)	
Off-axis_x_h	3.62	3.77	3.96	2.96	0.04 (−0.35–0.16)	
Off-axis_z_h	3.23	1.58	3.51	0.72	0.22 (−0.14–0.52)	

pre_ATT: duration of muscle activation; VL: Vastus Lateral; VM: Vastus Medial; O: muscle of the operated limb; H: muscle of the contralateral (healthy) limb; o: task executed with the operated limb; h: task executed with the contralateral (healthy) limb; env: maximum envelope of the sEMG signal; VGRF: vertical peak force; NPTS_Fy: number of vertical force peaks recorded at landing; ROM: range of motion of the knee joint; Off-axis: off-axis parameter.

**Table 3:** Characterization of biomechanical variables during the 5-repetition Sit-to-Stand test.

Variable	ACL		ACL-ALL			
	Median	IQR	Median	IQR	Effect Size	
duration_STS	8.25	1.73	7.76	3.72	0.04 (−0.13–0.12)	
dur_cycles_mean	1.19	0.25	1.21	0.31	0.18 (0.0014–0.34)	
peak_F_rise	541.6	112.41	452.87	134.8	**0.37 (0.21–0.53)**	**moderate**
mean_F_rise	333.6	92.2	304.65	45.25	0.20 (0.01–0.37)	
t_peak	0.14	0.03	0.14	0.03	0.18 (0.0036–0.35)	
max_env_OVL	0.2	0.15	0.1	0.26	0.27 (0.08–0.47)	
max_env_HVL	0.2	0.09	0.22	0.1	0.14 (−0.03–0.3)	
max_env_OVM	0.17	0.13	0.11	0.19	0.19 (0.0094–0.37)	
max_env_HVM	0.17	0.19	0.18	0.11	0.02 (−0.16–0.08)	
att_OVL	1.04	0.27	1.11	0.46	0.20 (0.02–0.38)	
att_HVL	1.08	0.27	1.19	0.24	0.26 (0.09–0.42)	
att_OVM	1.05	0.3	1.03	0.29	0.04 (−0.12–0.13)	
att_HVM	1.1	0.19	1.14	0.36	0.26 (0.08–0.44)	

duration_STS: duration of the entire task; dur_cycles_mean: average duration of the lift–seat cycles; peak_F: peak vertical force; rise: for ascent phase alone; mean_F: average vertical force during each cycle; t_peak: time required to reach this peak; max_env: peak of the envelope; VL: Vastus Lateral; VM: Vastus Medial; O: muscle of the operated limb; H: muscle of the contralateral (healthy) limb; att: duration of muscle activation.

**Table 4:** Characterization of biomechanical variables during the Countermovement Jump test with the change in direction of the knee.

Variable	ACL		ACL-ALL			
	Median	IQR	Median	IQR	Effect Size	
peak_envOVL_1o	0.486	0.214	0.356	0.264	0.32 (−0.02–0.64)	
peak_envOVL_2o	0.097	0.081	0.058	0.036	0.01 (−0.4–0.1)	
peak_envHVL_1o	0.466	0.306	0.311	0.281	0.17 (−0.17–0.41)	
peak_envHVL_2o	0.095	0.055	0.069	0.01	0.14 (−0.21–0.37)	
peak_envOVM_1o	0.51	0.15	0.342	0.109	0.30 (−0.06–0.61)	
peak_envOVM_2o	0.125	0.157	0.065	0.078	0.08 (−0.26–0.23)	
peak_envHVM_1o	0.561	0.254	0.493	0.261	**0.35 (0.05–0.66)**	**moderate**
peak_envHVM_2o	0.117	0.066	0.091	0.276	0.17 (−0.16–0.41)	
peak_envOVL_1h	0.5	0.166	0.401	0.214	0.34 (−0.0043–0.69)	
peak_envOVL_2h	0.105	0.106	0.109	0.042	0.03 (−0.39–0.15)	
peak_envHVL_1h	0.526	0.297	0.478	0.249	**0.40 (0.07–0.74)**	**moderate**
peak_envHVL_2h	0.13	0.13	0.097	0.156	0.30 (−0.04–0.63)	
peak_envOVM_1h	0.536	0.103	0.364	0.049	**0.38 (0.04–0.71)**	**moderate**
peak_envOVM_2h	0.178	0.206	0.095	0.184	**0.40 (0.04–0.76)**	**moderate**
peak_envHVM_1h	0.481	0.142	0.613	0.302	0.28 (−0.07–0.59)	
peak_envHVM_2h	0.212	0.089	0.095	0.398	0.09 (−0.26–0.26)	
onset_OVL_1o	1.46	0.4	1.39	0.44	0.22 (−0.13–0.51)	
onset_OVL_2o	0.68	0.62	0.45	0.56	0.18 (−0.16–0.44)	
onset_HVL_1o	1.45	0.61	1.24	0.07	0.09 (−0.3–0.29)	
onset_HVL_2o	0.3	0.46	0.36	0.53	0.09 (−0.26–0.26)	
onset_OVM_1o	1.45	0.4	1.45	0.33	0.01 (−0.4–0.1)	
onset_OVM_2o	0.55	0.46	0.58	0.54	0.14 (−0.22–0.37)	
onset_HVM_1o	1.4	0.43	1.14	0.35	0.09 (−0.27–0.26)	
onset_HVM_2o	28	0.72	0.25	0.29	0.15 (−0.19–0.38)	
onset_OVL_1h	1.21	0.66	1.35	0.24	0.17 (−0.17–0.41)	
onset_OVL_2h	0.47	0.4	0.71	0.13	0.12 (−0.27–0.33)	
onset_HVL_1h	1.25	0.43	1.27	0.5	0.01 (−0.41–0.1)	
onset_HVL_2h	0.66	0.6	0.9	0.55	0.09 (−0.3–0.27)	
onset_OVM_1h	1.25	0.49	1.35	0.38	0.01 (−0.39–0.09)	
onset_OVM_2h	0.59	0.51	0.62	0.27	0.01 (−0.4–0.11)	
onset_HVM_1h	1.32	0.42	1.38	0.34	0.12 (−0.24–0.31)	
onset_HVM_2h	0.95	0.66	0.19	0.76	**0.48 (0.16–0.8)**	**moderate**
GRF_1o	1408.853	429.844	1231.04	347.303	**0.45 (0.14, 0.78)**	**moderate**
GRF_2o	528.246	170.058	502.406	114.002	0.12 (−0.23–0.31)	
GRF_1h	1298.828	353.556	988.729	175.458	**0.53 (0.22, 0.86)**	**large**
GRF_2h	547.945	243.83	538.605	35.761	0.14 (−0.21–0.37)	

peak_env: sEMG envelope peak; O: muscle of the operated limb; H: muscle of the contralateral (healthy) limb; VL: Vastus Lateral; VM: Vastus Medial; 1: jumping phase; 2: foot rotation phase; o: task executed with the operated limb; h: task executed with the contralateral (healthy) limb; onset: duration of muscular activation; GRF: vertical force peak.

## Data Availability

The original contributions presented in this study are included in the article. Further inquiries can be directed to the corresponding author(s).
